# Association Between Performance on Visual Tests and Batting Performance Indicators in Highly Trained Baseball Players

**DOI:** 10.1177/00315125251395108

**Published:** 2025-11-27

**Authors:** Fabian Alberto Romero Clavijo, Maxime Trempe, Vanessa Bachir, Thomas Romeas

**Affiliations:** 1 Sports Studies Department, 4509 Bishop’s University, Sherbrooke, QC, Canada; 2 Institut national du sport du Québec, Montréal, QC, Canada; 3 École d’optométrie, 5622 Université de Montréal, Montréal, QC, Canada; 4 Department of Psychology, 7991 York University, Toronto, ON, Canada

**Keywords:** perceptual-cognitive skills, visual skills, expertise, talent identification, Bayesian statistics

## Abstract

Visual perception is crucial for successfully executing motor skills in interceptive sports like baseball. Understanding the contribution of visual skills (VS) to baseball performance is essential for talent selection and development. Some evidence suggests that performance on visual tests is predictive of batting performance, though these findings are not consistently replicated across studies. Therefore, the aim of this study is to associate a broad spectrum of visual performance indicators with a set of batting performance variables in highly trained baseball players by combining different methodological approaches used in previous studies. Forty-five highly trained male baseball players from the same club, aged between 15 and 19 years old (*mean* = 17.25), underwent a thorough battery of visual tests under standardized conditions. Twenty-one variables of VS were collected and associated with ten performance indicators, including game statistics, players’ ranking, age, years of practice, and position. Frequentist correlations and *t*-tests revealed that 17 out of the 210 associations (8.09%) reached our unadjusted threshold level and thus indicated a positive and statistically significant association. Bayesian analyses identified 34 associations (16.19%) that supported a positive association between VS and performance indicators, but only two of them (0.95%) revealed a moderate level of evidence in favor of the positive association. Therefore, this study provides limited support to the hypothesis that performance on visual tests predicts batting performance. The homogeneity of the sample and potential non-linear relations between visual and batting performance may account for these findings.

## Introduction

Interceptive motor tasks, for which the objective is to use the body or an implement to intercept a moving object, demand perceptual-cognitive skills (PCS) for successful execution (Williams & Weigelt, 2004). Specifically, successful baseball batting relies on the batter’s capacity to perceive visual cues such as the pitcher kinematics and the ball trajectory to identify the characteristics of the pitch and to plan the appropriate motor response (Burris et al., 2020; Kato & Fukuda, 2002). Most PCS studied in baseball rely on vision and are proposed to span a continuum ranging from fundamental (e.g., visual acuity) and low-level (e.g., stereoacuity) to high-level (e.g., visual attention) and cognitive functions (e.g., decision-making) (Hodges et al., 2021). Evidence suggests that visual skills (VS) at the beginning of the PCS continuum, correlate with batting performance (for a review, see Laby & Appelbaum, 2021). In light of this evidence, and given that VS training and testing programs are readily available, sports organizations have started to take an interest in VS measurements for talent identification, with the Major League Baseball Scouting Bureau even requiring visual performance testing for newly drafted players (Kirschen & Laby, 2021).

VS have typically been assessed using tests of sensorimotor and oculomotor functions, and the results have been used to assess their predictive value for baseball batting performance (Burris et al., 2018; Laby et al., 2018; Liu et al., 2020). In a study of static, kinetic, and dynamic visual acuity in 102 professional baseball players, Hoshina et al. (2013) revealed no performance differences in these skills among players of varying levels of performance. In contrast, Laby et al. (1996) investigated stereoacuity and contrast sensitivity in 387 major and minor league baseball players and identified significant differences in favor of major league players in four out of the 19 tested variables. These results were further corroborated by another study revealing positive correlations between visual acuity and plate discipline performance (Laby et al., 2019). In addition, in a study involving 450 professional baseball players, Laby et al. (2018) revealed that players with better eye-hand coordination, assessed on an LED board, presented better plate discipline, tended to play at higher competitive levels (i.e., major league instead of minor league), and enjoyed longer careers in the major leagues. However, the *r*^*2*^ values for these relationships ranged from 0.03 to 0.06, indicating small effect sizes. According to the authors, these small effect sizes were attributed to the fact that a multitude of factors contribute to successful batting performance in baseball.

In two separate studies, Burris et al. (2018) and Liu et al. (2020) collected sensorimotor data from 252 and 71 professional baseball players, respectively, competing in leagues ranging from rookie to major league (*mean* age = 22.4, *SD* = 3.2 years). Even though both studies conducted by the same research group investigated sensorimotor variables using the same battery of tests (i.e., Senaptec sensory station), their results were equivocal. Specifically, Burris et al. (2018) found that eye-hand coordination, reaction time, rapid shift focus, and visuo-spatial working memory were all associated with batting percentage, walks, and strikeouts. Conversely, Liu et al. (2020) found no association between sensorimotor performance and batting performance, with the latter assessed through plate discipline metrics. However, their analysis also included an assessment of oculomotor skills, which revealed that smooth pursuit accuracy and general oculomotor speed predicted the batter’s ability to decide which pitches to swing at based on their location in the strike zone. These mixed findings may reflect a weak relationship between VS and batting performance, or they may be attributed to confounding factors and methodological differences between the two studies. For example, Burris et al. (2018) included both batters and pitchers and used game statistics as performance indicators, while Liu et al. (2020) focused exclusively on batters and used plate discipline metrics.

To further investigate the impact of playing position on the association between performance on visual tests and baseball performance, studies have directly compared batters and pitchers, for whom the demands and training emphasis vary. While both batting and pitching involve a motor control component, batting is distinguished by its reliance on perceptual processing, as it is an anticipatory timing task that requires the processing of visual cues from the pitcher and the ball. In contrast, pitching is a self-paced task that primarily involves motor control to manipulate ball spin and speed (Gray & Sullivan, 2023; Molia et al., 1998). Klemish et al. (2018) compared nine sensorimotor variables between batters and pitchers playing at high school (*n* = 112), college (*n* = 85), and professional (*n* = 369) levels, using the same battery of tests employed in Burris et al. (2018) and Liu et al. (2020). They found that, at the professional level, batters presented better performance than pitchers in visual clarity and depth perception, whereas no differences were found between batters and pitchers at the high school and college levels. The authors attributed those differences to the amount of deliberate and specific practice that batters and pitchers require to achieve high levels of sport performance in their respective positions. The lack of differences by position in non-professional players was also found by Chen et al. (2021), who compared the eye-hand coordination and oculomotor functions as predictors of baseball batting performance between 44 national team-level players and 42 non-athletes. They found no significant performance differences in these variables across defensive positions (infielder, outfielder, pitcher, or catcher). In addition, eye-hand coordination and oculomotor functions displayed no significant correlation with years of practice. However, their results revealed that experienced players outperformed non-athletes in both eye-hand coordination and oculomotor functions.

In sum, studies investigating the same set of sensorimotor variables have identified some of these variables as predictors of batting performance, though these findings are not consistently replicated across studies. Regarding oculomotor skills, research indicates that these skills predict better plate discipline performance among professional players, but not when comparing by position or years of practice. Notably, the number of significant associations between VS and performance reported in these studies is relatively low compared to the total number of associations analyzed, with most exhibiting small effect sizes. Consequently, given the discrepancy between the results previously reported and the variety of methodological approaches used, further investigation is needed to clarify the association between performance on tests assessing a broad spectrum of VS and a range of batting performance indicators in baseball players. In the present study, we aimed to combine multiple methodological approaches to examine how a broad spectrum of VS measures relates to a set of batting performance variables in highly trained baseball players. Based on the inconsistency of prior evidence, we adopted a skeptical stance, and we expected no association between performance on visual tests and batting performance indicators.

## Method

### Participants

Forty-five highly trained (Tier 3; McKay et al., 2022) male baseball players, aged 15 to 19 years old (*mean* = 17.25, *SD* = 1.0), with an average of 11.15 years of playing experience (*SD =* 2.4, range 6–16 years), were recruited from the provincial-level program *Académie de Baseball du Canada*. Such a sample represents the entire population of players at that level and age in the province of Quebec, Canada. Therefore, data from all available players was collected (Lakens, 2022). On average, these players practiced baseball 20 h per week and competed in national and international events across North America. Ten players had previously represented the national team at various levels. At the time of data collection, several had committed—or were close to committing—to North American college programs. Of those players, twenty-four were batters and twenty-one were pitchers, with thirty-one being right-handed and fourteen left-handed. The research protocol (#2023-4706) was approved by the Université de Montréal research ethics board and athletes provided their informed written consent before participating.

### Data Collection

Data were collected at the local national sport institute under standardized conditions. Upon arrival at the testing facility, participants completed a custom questionnaire assessing their expertise in sports, visual health, dominant hand, years of baseball practice, and field position. Subsequently, they underwent in a quiet room a broad VS examination composed of a 60-min optometric evaluation and a 60-min visuomotor evaluation, completed in a counterbalanced order. The VS selected for these evaluations were chosen based on previous studies examining the predictive value of VS for batting performance (Burris et al., 2018; Chen et al., 2021; Klemish et al., 2018; Laby et al., 2018; Liu et al., 2020). Within the two evaluations, the VS tests were performed in the same order. Table 1 presents a description of each measured VS, ranging along a continuum from fundamental to cognitive skills. The optometric evaluation, conducted by an optometrist specialized in athletic populations, consisted of four tests, as follows.

**Table 1.** Spectrum of Visual Skills, Ranging Along a Continuum From Fundamental Visual Skills to Cognitive Skills (Adapted From Hodges et al. (2021))

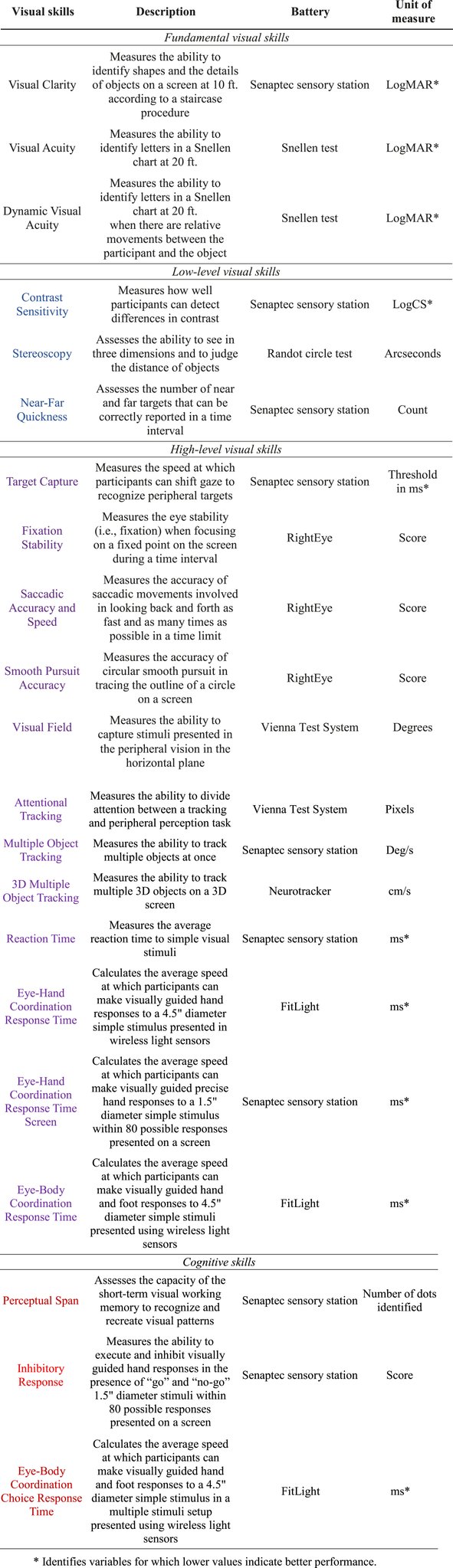

#### Static and Dynamic Visual Acuity

Visual acuity tests were performed with both eyes open using a Snellen chart projected 20 feet away. Participants were asked to read strings of five letters progressively until reaching the end of the 20/20 line. The criterion for advancing to the next line was accurately reading at least half of the letters on the current line. If participants were unable to do so, the visual acuity corresponding to the previous line was recorded as their result. In the static visual acuity test, participants were seated in a chair and kept their head still while reading. In the dynamic visual acuity test, participants were seated in the same chair but moved their head from side to side at a frequency of 2 Hz (i.e., 120 cycles per minute), guided by a metronome and monitored by the optometrist. The Snellen score was converted to a LogMAR score using the following formula:

$$LogMAR=round\left(\log\left(\frac{sden}{snum}\right),2\right)-\left(nlet\;x\;0.02\right)$$

where sden refers to the Snellen denominator value, snum refers to the Snellen numerator value, and nlet refers to the number of letters gained (+) or lost (−) (Tiew et al., 2020).

#### Stereoacuity

The Randot Circles stereo test (Stereo Optical Co., Inc., Chicago, USA) was used as a measure of stereoacuity (fine depth discrimination) and administered as described in the instruction guide. The participants held the Randot test book at 16 inches from their eyes while wearing polarized 3D glasses. They then identified one Wirt circle out of three that seemed to float forward. The participant’s stereo threshold was established as the last correctly identified level of the Wirt circle task. Possible scores on the Wirt circle task were 20, 25, 30, 40, 50, 70, 100, 140, 200, or 400 arc seconds.

#### Oculomotor Evaluation

This evaluation was performed using the RightEye Sensorimotor System^TM^ – Functional Vision EyeQ model (RightEye^®^, USA). In a quiet, private testing room, participants were seated in front of a height-adjustable table equipped with a Tobii I15 vision 15″ monitor fitted with a Tobii 90 Hz remote eye tracker and a Logitech (model Y-R0017) wireless keyboard and mouse. Oculomotor assessment consisted of five tests: circular smooth pursuit, horizontal smooth pursuit, horizontal saccades, vertical saccades, and fixation stability (Murray et al., 2019). In the pursuit tests, participants were instructed to follow a black dot with their gaze. In the saccade tests, they were instructed to move their eyes back and forth as quickly and accurately as possible between two dots. In the fixation stability test, participants were instructed to maintain their gaze in the center of a target. A general percentile rank score based on performance across the five tasks was automatically computed by the software for each participant.

The visuomotor evaluation was completed by one of the co-authors specialized in vision neurosciences and included four batteries of tests described as follows.

#### Sensorimotor Evaluation

A 25-min sensorimotor evaluation was conducted using the Senaptec Sensory Station (Senaptec Inc., USA). It included a battery of nine tasks designed to assess visuomotor skills which have demonstrated robust reliability in previous studies (Erickson et al., 2011; Wang et al., 2015; Zynda et al., 2024). The tasks measured visual clarity, contrast sensitivity, near-far quickness, target capture, multiple object tracking, reaction time (RT), perception span, eye-hand coordination response time screen, and go/no-go performance. A detailed description of this battery of tests, including an illustration of the tests, is provided in Figure S1 in the supplementary material.

#### Peripheral Vision

The Peripheral Perception Test from the Vienna Test System (Schuhfried, Austria) was used to assess peripheral field capacity and peripheral attention (Schuhfried, 2013). Participants were required to maintain focus for 7 minutes on a central task (using a joystick to track a moving dot) while responding (pressing a pedal) when vertical lines randomly flashed in the peripheral vision among distractors. This test measures tracking deviation while participants perform both central and peripheral tasks simultaneously.

#### Visual Tracking Speed

The Neurotracker^TM^ three-dimensional multiple object tracking (Neurotracker Inc., Canada) was used to assess the participants’ visual tracking speed during a 5-min test (Faubert & Sidebottom, 2012). During the test, participants wore active 3D glasses while seated 55 inches away from a 55-inch 3D TV screen, with their eyes aligned to the center of the display. Each trial required tracking four out of eight 3D balls randomly moving in a virtual space. In the first trial, the spheres moved at a starting speed of 68 cm/s, and then the speed varied between trials according to a 1-up 1-down staircase procedure. After 20 trials, the test provided the mean speed (in cm/s) of the last four trials, representing the visual tracking speed threshold.

#### Eye-Hand and Eye-Body Response Time

Adapted from Wilke et al. (2020), this custom-designed battery of tests was performed using the FitLight Trainer (FitLight Corp., USA). Three conditions were evaluated: eye-hand response time, eye-body response time, and eye-body choice response time. Each condition consisted of sixty trials, with each trial requiring a motor response to a visual stimulus randomly appearing in an eight-sensor setup positioned equidistantly in both the central and peripheral visual fields. Participants stood in front of the sensors, positioned at an elbow’s distance from each one. In the eye-hand response time condition, six sensors were placed on the wall and two on tripods, all above the waist level (see Figure S2 A in the supplementary material). Participants responded to a unique blue flashing light/stimulus using their preferred hand. The subsequent stimulus appeared only after a correct response was completed. In the eye-body response time condition, six sensors were placed above waist level— four on the wall and two on tripods— and two sensors were placed on the floor in front of each participant’s foot (see Figure S2 B in the supplementary material). Participants responded to a unique blue flashing stimulus using either their preferred hand (i.e., above-waist stimuli) or foot (i.e., floor stimuli). In the eye-body choice response time condition, the positioning of the sensors was identical to the eye-body response time condition, but participants responded using either their preferred hand or foot to a blue light appearing randomly among the eight sensors simultaneously with six distractors of other colors. The completion of three conditions lasted about 5 min. Response time was obtained in each condition.

#### Game Statistics

Batting-related game performance statistics from 24 matches played in provincial competitions during the competitive year were obtained using the GameChanger mobile app (GameChanger Media, Inc., USA). Based on previous studies (Burris et al., 2018; Liu et al., 2020; Müller & Fadde, 2016), yearly values of the following variables were sourced for each batter: games played, plate appearances, at bats, batting average, on-base percentage, slugging percentage, on-base plus slugging percentage, hits, bases on balls, and strikeouts. Following an initial analysis, multicollinearity was detected between games played, plate appearances, and at bats (r ≥ 0.89), and between batting average, slugging percentage, and on-base plus slugging percentage (r ≥ 0.88). Consequently, from these six variables, only at bats and batting average were retained, as they were deemed the most appropriate indicators of batting performance. This selection was based on the nature of each metric: while batting average reflects a player’s ability to perceive and make contact with the ball, slugging percentage and on-base plus slugging percentage emphasize power, which was not the primary focus of this analysis. Correlation values among all VS and among all batting performance indicators can be found in the supplementary material (Figures S3 and S4, respectively).

#### Players’ Ranking

Previous studies have highlighted the importance of subjective coach evaluations as complementary tools for talent identification (Abate Daga et al., 2024; Piggott et al., 2019). Therefore, we asked two batting coaches of the baseball team to report their subjective ordinal ranking of the batters based on overall performance on the following aspects: plate discipline, batting technique, situational hitting, and power generation. To assess inter-rater reliability, a weighted Cohen’s Kappa test was performed, revealing a correlation coefficient of 0.75, thus confirming substantial inter-rater agreement.

#### Other Performance Indicators

To further explore the association between VS and batting performance, additional performance variables were collected. Age, as an indicator of maturation (de la Rubia et al., 2020), was considered a variable associated with player expertise. Additionally, based on the hypothesis that more time of practice would result in better sport performance (Ericsson et al., 1993), years of baseball practice was used as another batting performance indicator. Lastly, as visual-cognitive demands are more important for batters than pitchers (Molia et al., 1998) and training activities vary between these positions (Klemish et al., 2018), playing position was used as a performance indicator by comparing performance on visual tests between these two playing positions.

### Statistical Analysis

First, variables where lower values indicated better performance were inverted for consistency and interpretation. To test data normality, Shapiro-Wilk tests were run for each variable. As most variables revealed non-normality (*p* < .05), one-tailed Spearman tests were run to correlate performance on each visual variable to game statistics, coaches’ ranking, age, and years of practice. Subsequently, for comparisons between batters and pitchers on each visual variable, both Mann-Whitney U tests and independent Student’s *t*-tests were performed. Given that both approaches produced comparable results, *t*-test outcomes were kept for reporting and interpretation. The software RStudio, Version 2023.09.0 (© 2009-2023), was used for data organization and frequentist statistical analysis. The alpha level was set to *p* < .05. Despite performing multiple correlations and *t*-tests (210 in total), the significance level was not adjusted, as methods like the Bonferroni correction would result in an overly conservative threshold, potentially concealing meaningful associations and increasing the risk of Type II errors (Emerson, 2020). The coefficient of determination (*r*^*2*^) and the eta-squared (*η*^*2*^) were used to report the effect size of the correlations and *t*-tests, respectively.

**Figure 1.**
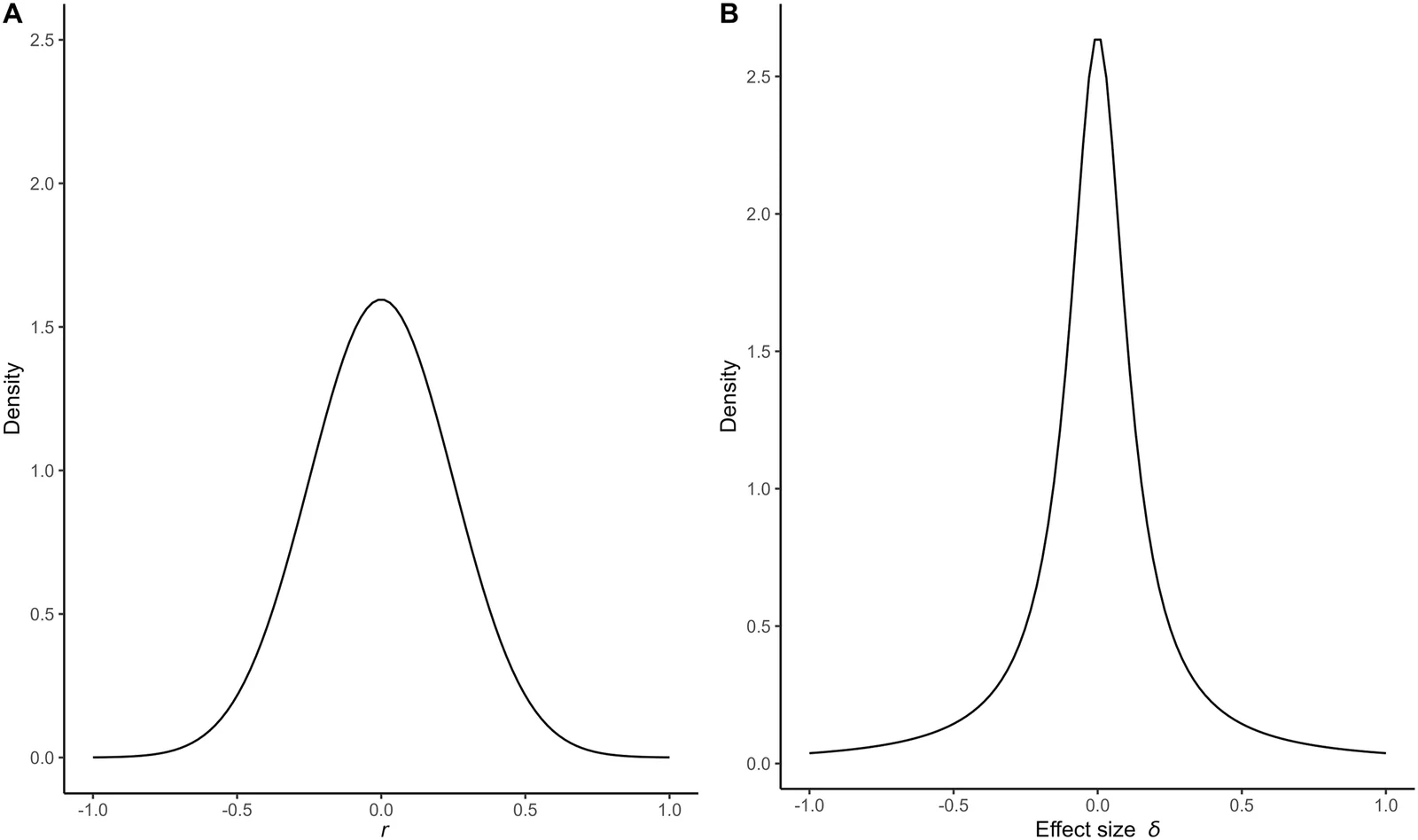
Distribution of Priors Chosen for Bayesian Correlations (A) and for the *t*-Tests (B)

One of the problems with the frequentist statistics described above is that they only provide support for the alternative hypothesis (*H*_*1*_). In other words, the alternative hypothesis is either accepted or rejected. Consequently, they hinder the possibility of evaluating the strength of evidence in favor of the null hypothesis (*H*_*0*_) (Rouder et al., 2009). In addition, frequentist statistics are very sensitive to sample size. In the context of experiments involving high-performance athletes, which tend to have small samples based on the relative rarity of the participants, statistical tests are often underpowered. As a result, whenever the null hypothesis is accepted, it is not always easy to determine whether this was the correct conclusion or whether the alternative hypothesis should have been accepted, provided that a larger sample size had been used. These problems can be alleviated by adopting a Bayesian approach in which the level of evidence favoring the null and alternative hypotheses can be quantified, without sample size being factored into the calculations (van de Schoot et al., 2015). This approach returns a Bayes factor, a continuous measure of evidence—ranging from anecdotal to extreme—in favor of one hypothesis over the other. A “moderate” level of evidence is generally considered a good level of support (see the different categories proposed by Wagenmakers et al. (2018) in Table S1 in supplementary material). Thus, we decided to complement the frequentist analyses described above with Bayesian correlations and t-tests computed using the JASP software (JASP Team, 2024). For the Bayesian correlations, *H*_*1*_ was defined as the presence of a correlation between our batting performance indicators and VS variables. A beta prior distribution was used, with a width parameter of 0.1 to reflect our skeptical stance, informed by posterior values and effect sizes reported in previous studies (Burris et al., 2018; Klemish et al., 2018; Laby et al., 2018, 2019; Liu et al., 2020). For Bayesian t-tests, *H*_*1*_ was defined as a difference between batters and pitchers. A Cauchy prior distribution was used, centered at 0 with a width parameter of 0.1 (Schmalz et al., 2023), consistent with the reasons above. Figure 1 illustrates the distribution of the priors used in our Bayesian analyses.

## Results

Descriptive statistics for each of the VS and batting performance variables are presented in Table S2 in the supplementary material.

### Frequentist Statistics

**Figure 2.**
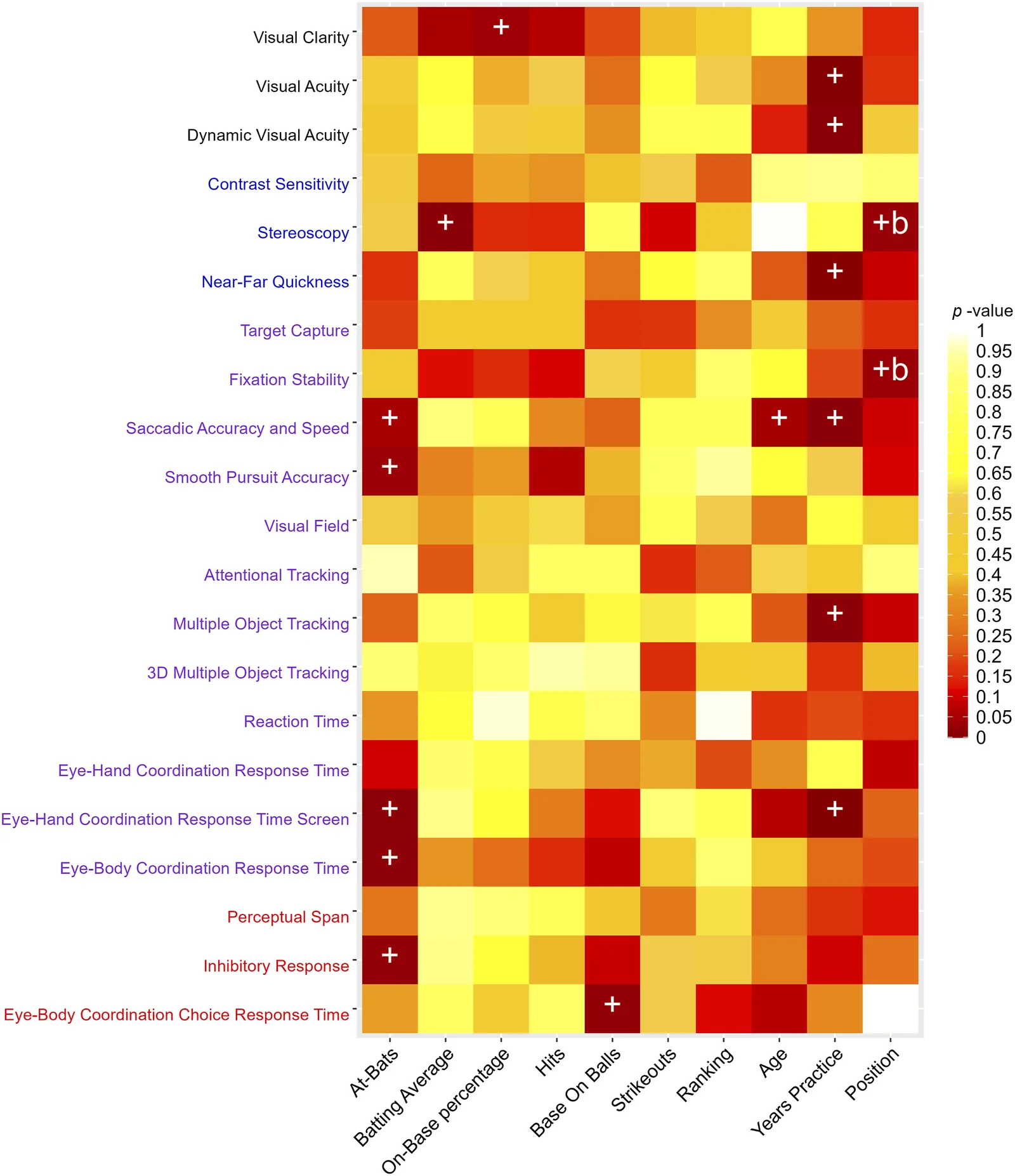
Heatmap of the *p*-Values of the Correlations Between VS and Batting Performance Indicators Along With the *p*-Values of the *t*-Tests Comparing VS by Position. When *p* < .05, the Symbols + Were Added to Indicate Significant Positive Correlations Between VS and Batting Performance. The Symbol + b Indicates a Significant Higher Performance in Batters Compared to Pitchers

**Figure 3.**
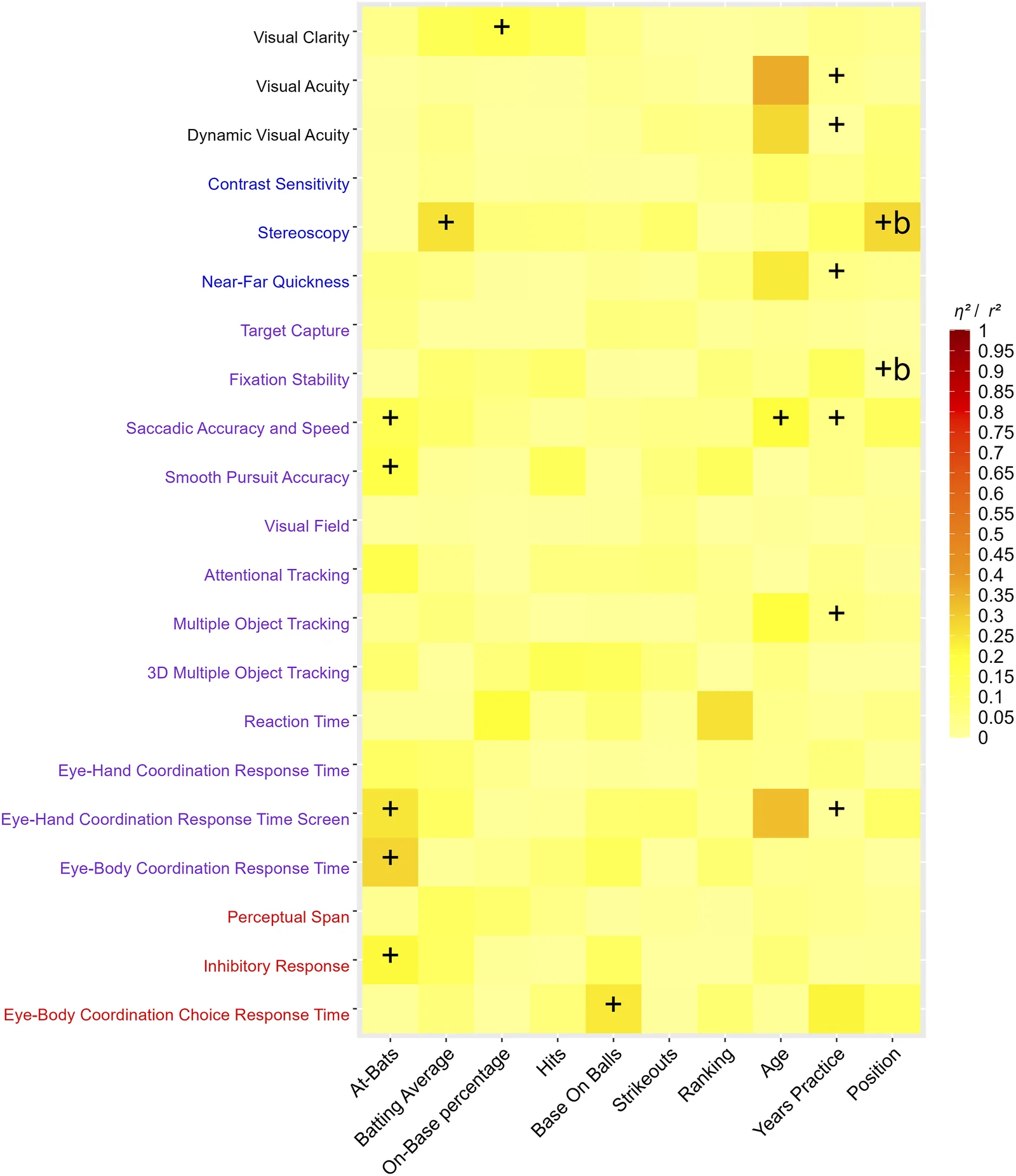
Heatmap of the Effect Sizes of the Correlations (r^2^) and *t*-Tests (η^2^). When *p* < .05, the Symbols + Were Added to Indicate Significant Positive Correlations Between VS and Batting Performance. The Symbol + b Indicates a Significant Higher Performance in Batters Compared to Pitchers

Figure 2 illustrates the *p*-values of the correlations between VS and batting performance indicators, along with the *p*-values of the *t*-tests comparing VS by position. Out of the 210 statistical tests computed (correlations and *t*-tests), 17 (8.09%) were significant. All of them corresponded to positive correlations, meaning that a higher VS score was associated with better batting performance or with batters outperforming pitchers. The effect sizes of all comparisons are illustrated in Figure 3. Overall, values ranged between 0 and 0.36, with most values (70%) revealing low effect sizes (i.e., <0.06) according to Cohen’s guidelines (Cohen, 2013). *p*-values and effect sizes for all correlations and *t*-tests between VS and performance indicators are presented in Tables S3 and S4 of the supplementary material.

### Bayesian Statistics

**Figure 4.**
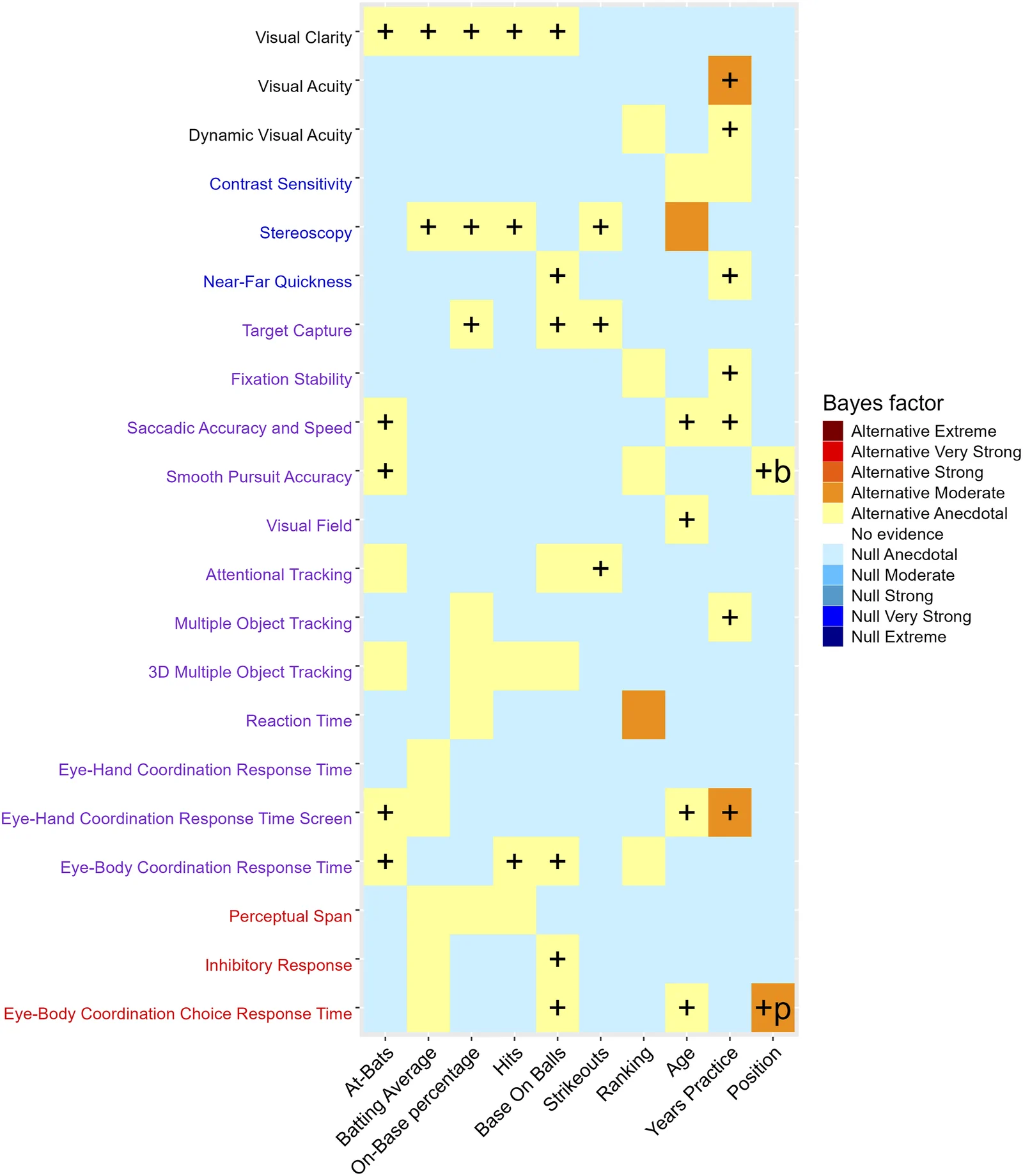
Heatmap of Bayes Factors of the Correlation and t-Tests Comparisons. The Symbol + was Added to Indicate a Positive Correlation Between VS and Batting Performance. The Symbols + b and + p Indicate a Higher Performance in Batters Compared to Pitchers, and Vice Versa for Pitchers Compare to Batters

Figure 4 illustrates the Bayes factors for the correlations between VS and batting performance indicators, along with the Bayes factors for the *t*-tests comparing VS by position. The results revealed that out of the 210 associations, 58 (27.62%) favored *H*_*1*_, and 152 associations (72.38%) favored *H*_*0*_. Among the associations favoring *H*_*1*_, 34 (16.19%) supported a positive relation between VS and batting performance (i.e., a higher VS score is associated with better batting performance). Out of these, two associations (0.95%) moderately supported *H*_*1*_, while the remaining 32 (15.24%) revealed anecdotal support for *H*_*1*_. With regard to the 152 associations supporting *H*_*0*_, all of them revealed an anecdotal level of support. Bayes factor for all correlations and *t*-tests between VS and performance indicators are presented in Table S5 of the supplementary material.

## Discussion

This study investigated the association between a broad spectrum of visual measures and a set of performance indicators in highly trained baseball players. Based on the large discrepancy of results currently existing in the literature, we adopted a skeptical stance and expected no association between performance on visual tests and batting performance indicators. Overall, frequentist Spearman correlations and *t*-tests revealed only 17 significant positive associations (8.09%) between performance on visual tests and batting performance indicators. Conversely, 193 associations/comparisons (91.91%) were either non-significant or negatively associated.

Importantly, interpretation of these results must be done in light of our decision not to adjust the alpha level to account for the multiple comparisons performed. As a result, 5% of the associations were expected to be significant by chance alone, representing false positives. This proportion closely matches the percentage of significant associations we reported, thereby casting doubt on their validity. This concern is further supported by the observation that the positive and significant associations were spread across eight different VS. If a VS were truly predictive of batting performance, one would expect it to be correlated with multiple batting performance indicators. Taken together, our results support the hypothesis that there is no association between VS and batting performance.

While it could be argued that our frequentist statistics were underpowered to detect differences having the observed effect size, the Bayesian analyses, which are not sensitive to sample size, led to a similar conclusion. We observed evidence in favor of the alternative hypothesis (i.e., a positive relation between VS and batting performance; *H*_*1*_) in 34 associations (16.19%). Of them, 32 associations (15.24%) involving 15 VS showed anecdotal support for *H*_*1*_, suggesting a low probability of them reflecting true effects. Only two associations (0.95%) involving two VS (visual acuity and eye-hand coordination response time screen) moderately supported *H*_*1*_. Our confidence in these two variables representing a genuine predictor of batting performance is, however, dampened by the observation that they represent two isolated cases within a broader set of evidence supporting no association or anecdotal support for *H*_*1*_. For instance, in the case of visual acuity, it was the only association revealing moderate support for *H*_*1*,_ while the other nine associations provided anecdotal support for *H*_*0*_. With regard to the eye-hand coordination response time screen, six of the other nine associations with batting performance indicators revealed anecdotal support for *H*_*0*_, while three revealed anecdotal support for *H*_*1*_. In sum, the scarce support for *H*_*1*_, the inconsistent patterns throughout the spectrum of analysis, and the high number of associations anecdotally supporting the null hypothesis (152 associations; 72.38%) all point toward an absence of positive relation between VS and the batting performance indicators in our sample of athletes.

Previous studies assessing a narrow range of VS often identified only a few VS as significant predictors of batting performance. For example, Burris et al. (2018) found that only six (15.0%) of the analyzed variables were significant predictors of batting performance, a result similar to the five (10.4%) variables across 12 regression models identified by Liu et al. (2020). Similarly, Klemish et al. (2018) found significant differences between the VS of pitchers and batters in only 7.4% of the comparisons. These consistently low percentages of significant associations suggest, at best, an unclear relationship between vision and batting performance. An important consideration when evaluating this evidence is not only its limited number but also its inconsistency. Variables identified as significant predictors in one study are often not corroborated in others. For example, perceptual span, reaction time, near-far quickness, and target capture have been associated with game statistics such as batting average, walks, and strikeouts (Burris et al., 2018). However, our results revealed negative or no association for the same variables. Furthermore, while visual acuity was reported to correlate positively with plate discipline performance in professional players (Laby et al., 1996, 2019), such a result was not replicated in a later study by Liu et al. (2020), who instead identified smooth pursuit accuracy and general oculomotor speed as significant predictors of plate discipline performance. While our study did not include measures of plate discipline specifically, all our performance indicators (some being on-field indicators) revealed no relation with VS. Overall, the limited and inconsistent findings across studies further reinforce the assumption that there may be no relation between VS and batting performance.

Considering the results of this study alongside previous research, we question why performance on visual tests often fails to correlate with batting performance indicators. This may be explained by the fact that tests conducted in well-controlled laboratory conditions, such as those used in this study, do not accurately represent the multi-sensory information of the sport environment, including movement, noise, and variable lighting conditions. This might have impacted the participants’ performance, as previous studies have shown that performance on perceptual-cognitive tests tends to worsen in silent conditions compared to conditions with noisy environments (Wilkins, 2024b). More specifically, generic laboratory-based tests do not reflect the actual visual demands of batting. For example, testing tasks aiming at assessing visual clarity typically requires participants to identify a static object (such as a letter or shape) without temporal constraints and to provide a simple declarative response. This does not fully capture the visual demands of a sport context like baseball batting, which requires players to extract information from multiple dynamic visual cues—such as the ball’s seams, the pitcher’s finger placement, and wrist movement (Liu et al., 2020)—all under strict time constraints. Similarly, the eye-body coordination tests measure response time by requiring participants to press a light sensor with a hand or foot in response to a simple stimulus. However, the eye-body coordination demands in baseball batting are far more complex, involving the processing of visual information to guide a complex motor response that engages multiple joints across both the upper and lower body (Gray, 2020). The integrative and context-specific nature of these perceptual-cognitive demands may therefore be poorly captured by isolated visual tests. This is supported by evidence from various domains, including sport performance, which suggests that integrative phenomena cannot be fully understood by merely isolating and summing independent components (Araújo & Davids, 2016; Hadlow et al., 2018; Weiss et al., 1967). In sum, the mismatch between perceptual-cognitive demands of visual tests and baseball batting may explain the lack of associations observed in the present study and in others. In this context, the evidence provides little support for the necessity of an examination of visual performance involving multiple tests during the Major League Baseball draft (Kirschen & Laby, 2021), beyond its usefulness in verifying ocular health.

Another factor that could explain the lack of association between VS and batting performance is that above-average visual performance may not be necessary for success in interception tasks. For example, research indicates that even with certain levels of induced blur, good performance is maintained in interceptive sports like cricket (Mann et al., 2010) and boxing (Limballe et al., 2024). More specifically, evidence has revealed that batting performance declines only when an over-refraction of +3.00 diopters is induced, resulting in a drop in visual acuity to 6/60 (Mann et al., 2007). Thus, it is possible that beyond a certain level of visual performance, its relation with batting performance is no longer linear. This nonlinear relation suggests that once a certain threshold of VS is reached, further improvements may not translate into better batting performance, a hypothesis that is aligned with the limited associations observed in the current study. Moreover, while vision plays a crucial role in successful baseball batting, the influence of many other factors (Laby et al., 2018) likely contributes to the limited positive associations observed in this study. For example, batting performance is affected by factors such as the batter’s fatigue, strength, and anthropometrics (Kohn et al., 2024), the stage of the competition (Conforti et al., 2021), and even the environmental temperature during the match (Huang et al., 2021). Additionally, game score and pitcher-batter count can impact batting performance as they influence the likelihood of receiving specific pitches, which in turn affects the batter’s perceptual performance (Gray & Cañal-Bruland, 2018). In summary, multiple factors that contribute to batting performance help explain why different levels of visual performance are not consistently associated with it.

It is noteworthy that while previous studies included participants across a broad age range (14 to 40 years) and varying competition levels (from high school to professional players, including rookies to major leaguers), the present study used a more homogenous group (high school students aged 15 to 19, all participating in the same baseball development program). Additionally, prior research often collected data over multiple years, whereas our study was limited to a single season. These differences in sample composition and methodology in early studies likely resulted in a broader range of values, which may have facilitated stronger associations between VS and batting performance. However, according to the results of the present study, when evaluating players within a relatively homogeneous group to identify the top performers, such as in the MLB draft, VS appears to have limited utility. This finding highlights a potential weakness of VS testing for talent identification in real-world scenarios.

Based on evidence from this and previous studies, we suggest that visual tests measuring sport-specific perceptual-cognitive demands of baseball batting could provide a better basis for linking perceptual-cognitive and baseball batting performance (Kalén et al., 2021; Spitz et al., 2018). In that context, the classification framework for perceptual-cognitive training in sport proposed by Hadlow et al. (2018) offers a useful foundation for developing sport-specific perceptual-cognitive tests. The framework identifies three key factors that influence performance and should be considered when designing such tests: targeted perceptual function, stimulus correspondence, and response correspondence. One potential option to measure the anticipatory capacity of baseball batters is the pitch recognition test (Moore & Müller, 2014). This test assesses the baseball batters’ ability to identify pitch type and location. However, previous evidence has also found limited correlation between performance on the pitch recognition test and batting game statistics (Fadde, 2016; Müller & Fadde, 2016). One possible explanation for the lack of association is that stimuli, often presented on digital screen projections (i.e., cellphones, tablets, or computers), might not correspond to the demands of the batting task. These presentation methods might fail to accurately replicate key aspects of the task, such as pitcher and ball size, movement speed, and distance. A promising technology that could improve the realism of pitch recognition tasks is extended reality, such as virtual reality and immersive 360° videos (Wilkins, 2024a). That technology allows users to perceive and interact with computer-simulated or real environments displayed on devices like CAVE systems or head-mounted displays (Kittel et al., 2024). Compared to traditional screen projections, immersive 360° videos have shown significant potential for enhancing stimulus correspondence applications in sports (Loiseau Taupin et al., 2023; Pagé et al., 2019). This represents a promising direction for future studies aimed at developing reliable perceptual-cognitive tests, which could then be used to support the monitoring of player development and enhance scouting processes.

## Conclusion

The findings of this study suggest that VS performance is not associated with batting performance, thus limiting its effectiveness for talent identification. Thus, further research should explore the associations between VS tested in a sport-specific context and work toward developing valid perceptual-cognitive tests that assess batting-specific perceptual demands.

## Supplemental Material

Supplemental Material - Association Between Performance on Visual Tests and Batting Performance Indicators in Highly Trained Baseball Players Supplemental Material for Association Between Performance on Visual Tests and Batting Performance Indicators in Highly Trained Baseball Players by Fabian Alberto Romero Clavijo, Maxime Trempe, Vanessa Bachir, Thomas Romeas in Perceptual and Motor Skills

## Data Availability

The data that support the findings of this study are available from the corresponding author upon reasonable request.*
