# Supplementary material for: Association Between Performance on Visual Tests and Batting Performance Indicators in Highly Trained Baseball Players
Source: Percept Mot Skills. 2025 Nov 27;133(5):1567–90. doi: 10.1177/00315125251395108 (PMC13569889; doi:10.1177/00315125251395108)
Supplement: Supplementary file 1 [file sj-pdf-1-pms-10.1177_00315125251395108.pdf]

Supplementary material

Table S1. Categories for interpreting the Bayes factor (Jeffreys, 1961; Wagenmakers et al., 2018)

Bayes factor	Evidence category
> 100	Extreme evidence for H_1
30 - 100	Very strong evidence for H_1
10 - 30	Strong evidence for H_1
3 - 10	Moderate evidence for H_1
1 - 3	Anecdotal evidence for H_1
1	No evidence
1/3 - 1	Anecdotal evidence for H_0
1/10 - 1/3	Moderate evidence for H_0
1/30 - 1/10	Strong evidence for H_0
1/100 - 1/30	Very strong evidence for H_0
< 1/100	Extreme evidence for H_0

Table S2. Descriptive statistics for each of the VS and batting performance variables or batters

**Variable**	***Min***	***Max***	***Mean***	***SD***
Visual Clarity (LogMAR)	0.21	0.69	0.47	0.11
Visual Acuity (LogMAR)	-0.18	0.14	0.01	0.09
Dynamic Visual Acuity (LogMAR)	20	40	26.05	8.09
Contrast Sensitivity (LogCS)	1.2	2	1.53	0.25
Stereoscopy (Arc seconds)	-0.2	-0.12	-0.14	0.03
Near-Far Quickness (Count)	15	28	22.17	3.85
Target Capture (Threshold in ms)	150	225	190	18.85

Fixation Stability (Score)	69	99	91.54	8.31
Saccadic Accuracy and Speed (Score)	65	93	85.91	8.3
Smooth Pursuit Accuracy (Score)	45	82	67.5	11.44
Visual Field (Degrees)	289	402	343.25	27.25
Attentional Tracking (Pixels)	173.1	190.8	183.75	4.62
Multiple Object Tracking (Deg/s)	32	61	48.42	10.28
3D Multiple Object Tracking (cm/s)	6.05	11.95	8.07	1.47
Reaction Time (ms)	364	648	522.5	84.79
Eye-hand Coordination Response Time (ms)	45.03	160.48	90.75	29.37
Eye-hand Coordination Response Time screen (ms)	547.4	685.62	607.61	39.04
Eye-body Coordination Response Time (ms)	530.3	814.8	701.12	75.99
Perceptual Span (Number of dots identified)	0	14	5.22	4.6
Inhibitory Response (Score)	1003.72	1171.23	1095.67	41.14
Eye-body Coordination Choice Response Time (ms)	886.48	1050.04	984.68	43.93
At Bats (Total)	6	46	27.95	9.13
Batting Average	0.17	0.46	0.31	0.09
On-Base %	0.29	0.57	0.43	0.08
Hits (Total)	1	15	8.74	3.84
Base On Balls (Total)	1	10	5.41	2.85
Strikeouts (Total)	1	14	7.83	3.59

Age (years)	15.04	19.62	17.14	1.13
Years Of Practice (years)	6	16	11.08	2.45

Table S3. P-values from frequentist correlations and t-tests between VS and performance indicators.

**Visual Skills**	**At-Bats**	**Batting Average**	**On-Base percentage**	**Hits**	**Base On Balls**	**Strikeouts**	**Ranking**	**Age**	**Years Practice**	**Position**
Visual Clarity	0.22	0.05	0.04	0.07	0.2	0.39	0.45	0.78	0.34	0.15
Visual Acuity	0.5	0.7	0.38	0.59	0.26	0.71	0.57	0.31	0	0.16
Dynamic Visual Acuity	0.41	0.76	0.53	0.5	0.33	0.78	0.79	0.13	0.01	0.53
Contrast Sensitivity	0.54	0.24	0.37	0.34	0.4	0.58	0.22	0.91	0.92	0.88
Stereoscopy	0.56	0.01	0.15	0.15	0.82	0.11	0.46	1	0.78	0.03
Near-Far Quickness	0.16	0.8	0.59	0.42	0.27	0.69	0.86	0.22	0.01	0.09
Target Capture	0.19	0.45	0.45	0.45	0.16	0.17	0.32	0.5	0.23	0.16
Fixation Stability	0.49	0.12	0.16	0.11	0.6	0.47	0.86	0.67	0.19	0.03
Saccadic Accuracy and Speed	0.05	0.89	0.79	0.32	0.23	0.8	0.8	0.05	0.01	0.1
Smooth Pursuit Accuracy	0.03	0.3	0.35	0.06	0.39	0.84	0.94	0.68	0.58	0.11
Visual Field	0.55	0.36	0.51	0.61	0.36	0.8	0.58	0.27	0.72	0.44
Attentional Tracking	0.96	0.22	0.55	0.83	0.83	0.16	0.22	0.6	0.45	0.9
Multiple Object Tracking	0.23	0.84	0.73	0.44	0.64	0.62	0.79	0.22	0.01	0.09
3D Multiple Object Tracking	0.88	0.63	0.86	0.95	0.93	0.16	0.45	0.5	0.16	0.39
Reaction Time	0.34	0.66	0.97	0.75	0.87	0.31	0.99	0.17	0.2	0.16
Eye-Hand Coordination Response Time	0.11	0.87	0.75	0.55	0.32	0.37	0.2	0.33	0.77	0.08
Eye-Hand Coordination Response Time Screen	0.02	0.91	0.65	0.29	0.12	0.89	0.79	0.07	0	0.23
Eye-Body Coordination Response Time	0.02	0.34	0.25	0.15	0.08	0.48	0.88	0.49	0.25	0.2
Perceptual Span	0.27	0.92	0.89	0.8	0.41	0.28	0.61	0.26	0.17	0.12
Inhibitory Response	0.02	0.91	0.68	0.39	0.09	0.58	0.56	0.29	0.1	0.27
Eye-Body Coordination Choice Response Time	0.36	0.84	0.48	0.84	0.02	0.58	0.12	0.07	0.31	1

Table S4. Effect size from frequentist correlations (r^2) and t-tests (η^2) between VS and performance indicators.

**Visual Skills**	**At-Bats**	**Batting Average**	**On-Base percentage**	**Hits**	**Base On Balls**	**Strikeouts**	**Ranking**	**Age**	**Years Practice**	**Position**
Visual Clarity	0.04	0.15	0.17	0.13	0.04	0.01	0.00	0.01	0.04	0.03
Visual Acuity	0.00	0.02	0.01	0.00	0.03	0.02	0.00	0.36	0.03	0.01
Dynamic Visual Acuity	0.00	0.04	0.00	0.00	0.02	0.05	0.04	0.27	0.00	0.07
Contrast Sensitivity	0.00	0.03	0.01	0.01	0.00	0.00	0.03	0.08	0.04	0.08
Stereoscopy	0.00	0.25	0.06	0.07	0.05	0.09	0.00	0.03	0.11	0.27
Near-Far Quickness	0.06	0.04	0.00	0.00	0.02	0.02	0.06	0.24	0.04	0.03
Target Capture	0.05	0.00	0.00	0.00	0.06	0.05	0.01	0.03	0.02	0.00
Fixation Stability	0.00	0.08	0.06	0.09	0.00	0.00	0.06	0.03	0.12	0.01
Saccadic Accuracy and Speed	0.15	0.09	0.04	0.01	0.03	0.04	0.04	0.20	0.04	0.12
Smooth Pursuit Accuracy	0.18	0.02	0.01	0.13	0.01	0.06	0.12	0.00	0.04	0.01
Visual Field	0.00	0.01	0.00	0.00	0.01	0.04	0.00	0.02	0.00	0.02
Attentional Tracking	0.16	0.04	0.00	0.05	0.05	0.06	0.03	0.00	0.04	0.00
Multiple Object Tracking	0.03	0.06	0.02	0.00	0.01	0.01	0.03	0.20	0.05	0.03
3D Multiple Object Tracking	0.08	0.01	0.07	0.15	0.12	0.06	0.00	0.04	0.00	0.00
Reaction Time	0.01	0.01	0.20	0.03	0.08	0.01	0.26	0.03	0.02	0.04
Eye-Hand Coordination Response Time	0.10	0.08	0.03	0.00	0.01	0.01	0.04	0.03	0.06	0.01
Eye-Hand Coordination Response Time Screen	0.25	0.10	0.01	0.02	0.08	0.09	0.03	0.33	0.01	0.10
Eye-Body Coordination Response Time	0.28	0.01	0.03	0.07	0.13	0.00	0.08	0.02	0.03	0.00
Perceptual Span	0.02	0.11	0.08	0.04	0.00	0.02	0.00	0.04	0.03	0.02
Inhibitory Response	0.21	0.11	0.01	0.01	0.10	0.00	0.00	0.07	0.01	0.01
Eye-Body Coordination Choice Response Time	0.01	0.06	0.00	0.07	0.24	0.00	0.08	0.01	0.22	0.10

Table S5. Bayes Factor from frequentist correlations and t-tests between VS and performance indicators.

**Visual Skills**	**At-Bats**	**Batting Average**	**On-Base percentage**	**Hits**	**Base On Balls**	**Strikeouts**	**Ranking**	**Age**	**Years Practice**	**Position**
Visual Clarity	1.18	1.62	1.75	1.79	1.10	0.73	0.70	0.86	0.68	0.80
Visual Acuity	0.73	0.77	0.74	0.73	0.76	0.81	0.73	0.75	7.65	0.82
Dynamic Visual Acuity	0.80	0.77	0.82	0.77	0.80	0.76	1.25	0.72	1.50	0.87
Contrast Sensitivity	0.76	0.79	0.74	0.76	0.73	0.72	0.81	1.11	1.68	0.94
Stereoscopy	0.72	1.92	1.01	1.00	0.80	1.05	0.72	5.09	0.70	0.87
Near-Far Quickness	0.97	0.90	0.73	0.73	1.05	0.89	0.96	0.89	2.88	0.89
Target Capture	0.76	0.84	1.03	0.87	1.43	1.08	0.73	0.70	0.69	0.99
Fixation Stability	0.72	0.79	0.75	0.73	0.73	0.72	1.07	0.70	1.16	0.95
Saccadic Accuracy and Speed	1.01	0.81	0.73	0.77	0.91	0.95	0.81	1.58	2.36	0.96
Smooth Pursuit Accuracy	1.05	0.72	0.74	0.85	0.72	0.92	1.14	0.68	0.70	1.13
Visual Field	0.72	0.79	0.76	0.80	0.75	0.80	0.70	1.10	0.69	0.80
Attentional Tracking	1.52	0.91	0.72	0.86	1.13	1.35	0.76	0.70	0.68	0.87
Multiple Object Tracking	0.73	0.87	1.12	0.74	0.87	0.72	0.89	0.79	2.01	1.00
3D Multiple Object Tracking	1.07	0.76	1.08	1.09	1.21	0.78	0.69	0.68	0.82	0.79
Reaction Time	0.73	0.76	1.04	0.74	0.76	0.75	3.65	0.74	0.78	0.81
Eye-Hand Coordination Response Time	0.76	1.12	0.84	0.78	0.81	0.79	0.95	0.82	0.70	0.90
Eye-Hand Coordination Response Time Screen	1.11	1.06	0.81	0.75	0.98	0.91	0.73	1.06	8.22	0.83
Eye-Body Coordination Response Time	2.06	0.79	0.82	1.22	1.10	0.75	1.06	0.70	0.75	0.81
Perceptual Span	0.79	1.43	1.07	1.07	0.75	0.85	0.69	0.94	0.92	0.93
Inhibitory Response	0.98	1.04	0.75	0.72	1.19	0.72	0.70	0.77	0.83	0.78
Eye-Body Coordination Choice Response Time	0.74	1.10	0.75	0.91	1.14	0.75	0.72	1.07	0.71	3.87

Visual Clarity	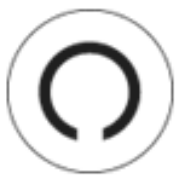	Measures the ability to identify shapes and the details of objects in a screen at 10 ft according to a staircase procedure
Contrast Sensitivity	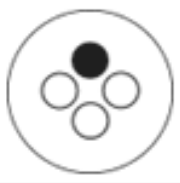	Measures how well participants can detect differences in contrast
Near-Far Quickness	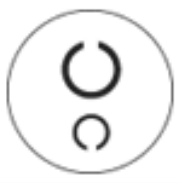	Assesses the number of near and far targets that can be correctly reported in a time interval
Target Capture	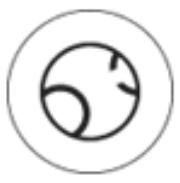	Measures the speed at which participants can shift gaze to recognize peripheral targets
Multiple Object Tracking	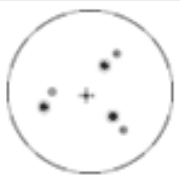	Measures the ability to track multiple objects at once
Reaction Time	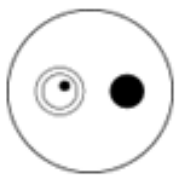	Measures the average reaction time to simple visual stimuli
Eye-Hand Coordination Response Time Screen	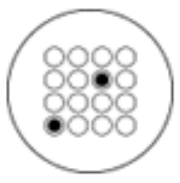	Calculates the average speed at which participants can make visually guided precise hand responses to a 1.5" diameter simple stimulus within 80 possible responses presented on a screen
Perceptual Span	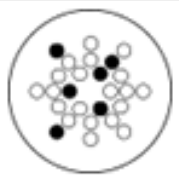	Assesses the capacity of the short-term visual working memory to recognize and recreate visual patterns
Inhibitory response	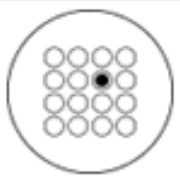	Measures the ability to execute and inhibit visually guided hand responses in the presence of "go" and "no-go" 1.5" diameter stimuli within 80 possible responses presented on a screen

Figure S1. Illustration and description of the nine tasks involved in the sensorimotor evaluation.

A B

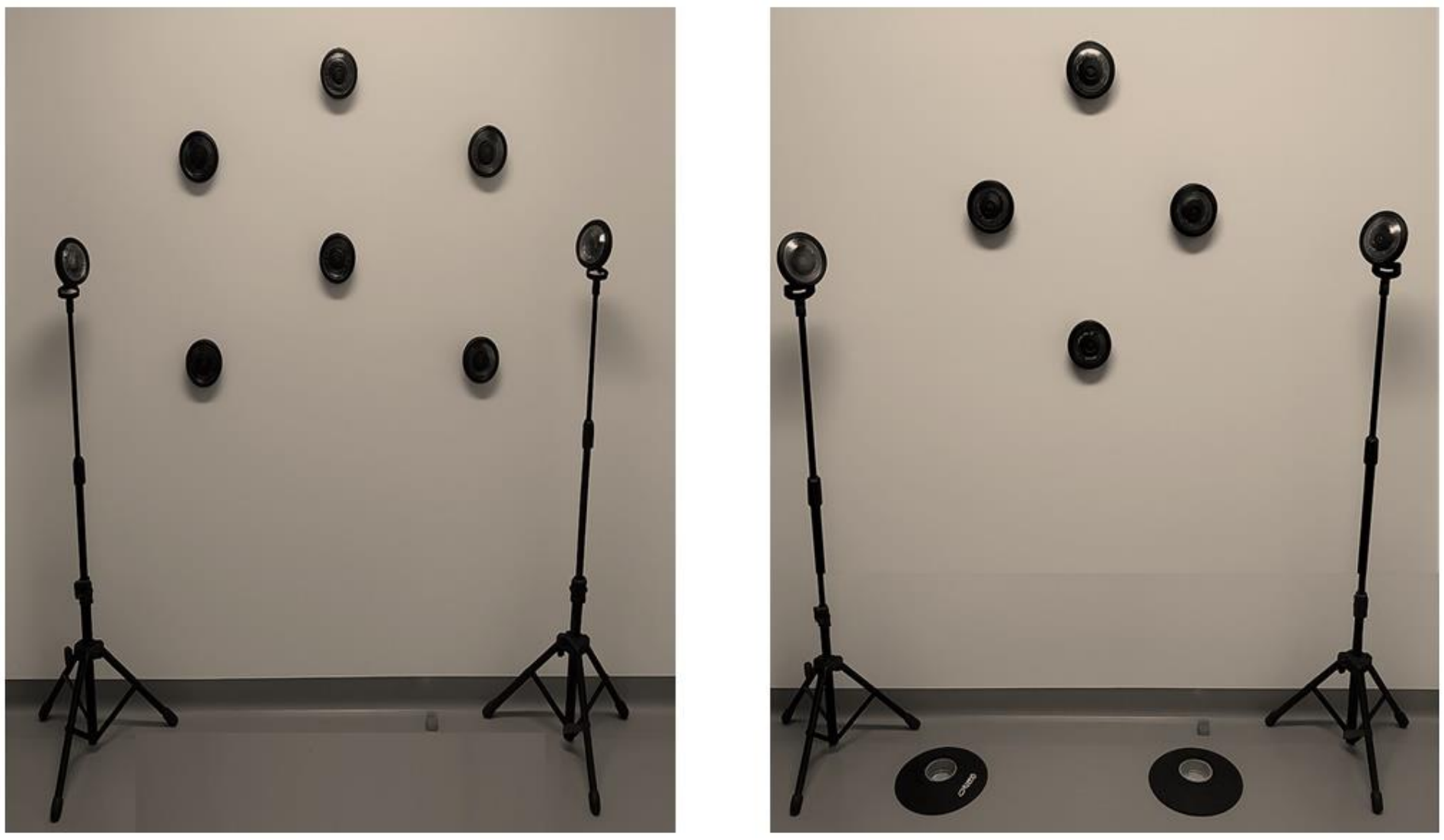

Figure S2. Illustration of the experimental setup for the eye-hand response time test (A), and the eye-body response time and eye-body choice response time tests (B).

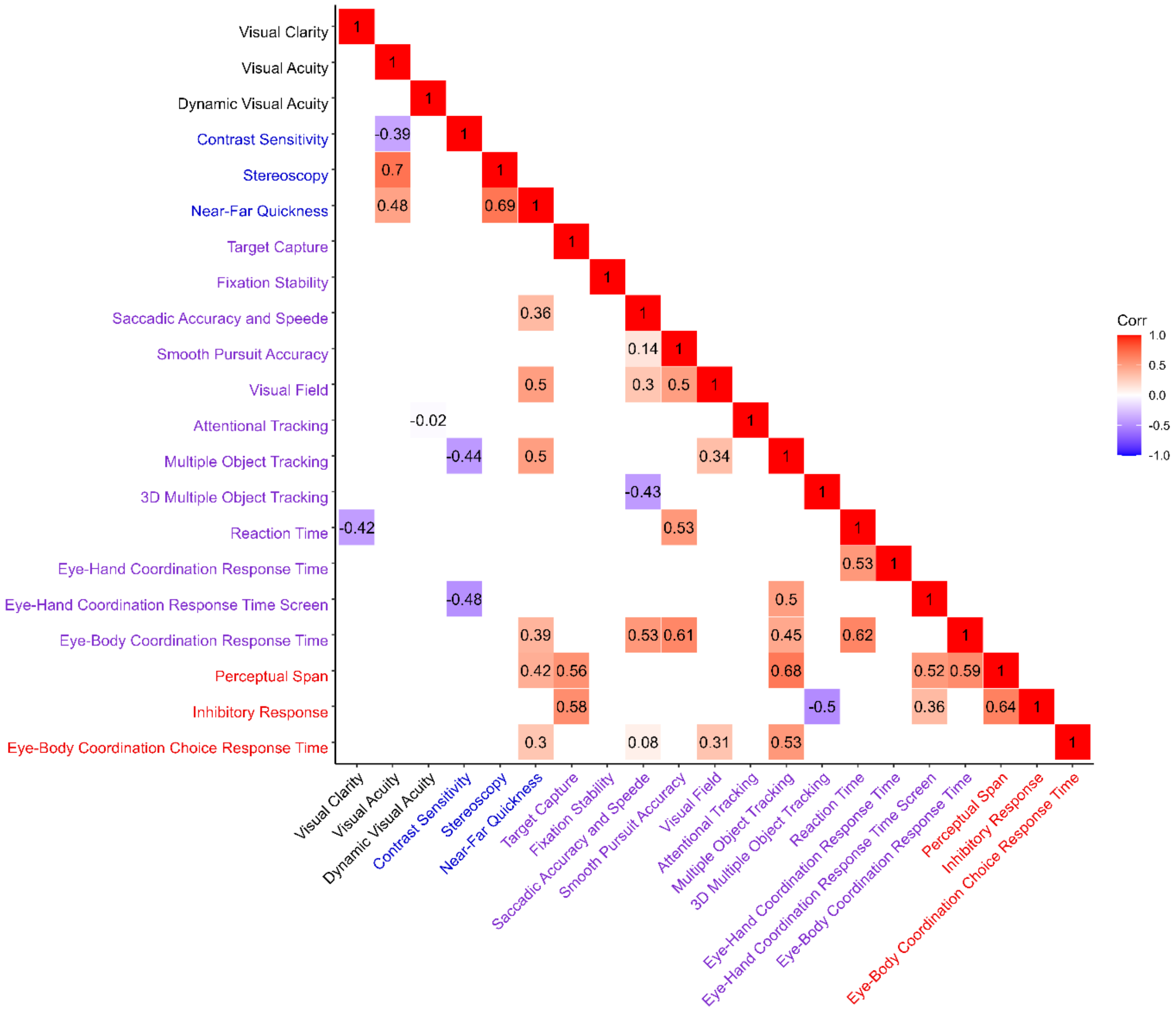

Figure S3. Correlation heatmap among VS. Only statistically significant correlations ($p < .05$) are displayed.

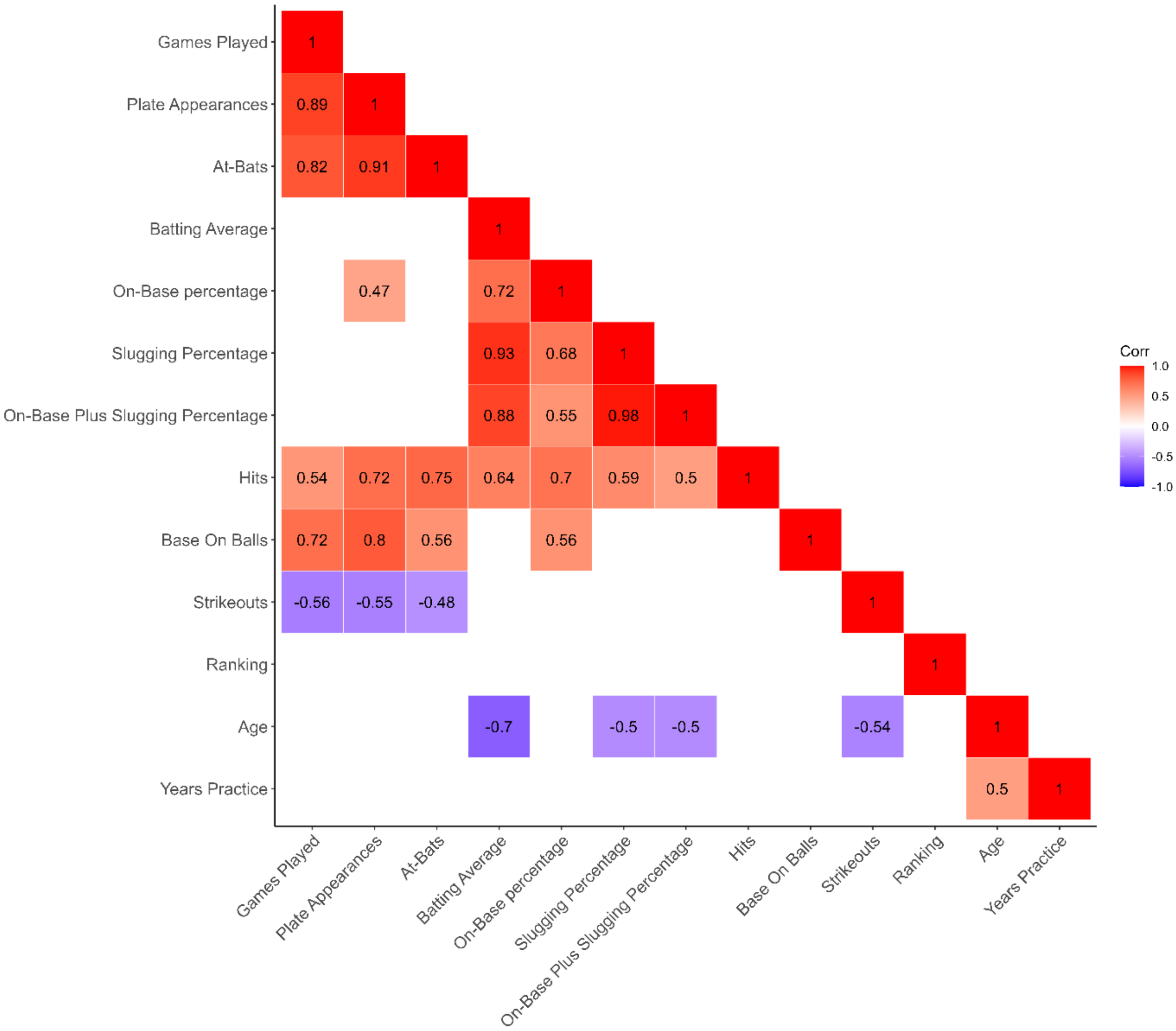

Figure S4. Correlation heatmap among batting performance indicators, including those removed due to multicollinearity. Only statistically significant correlations ($p < .05$) are displayed.
